# Do symptoms moderate the association between participation and executive functions outcomes among people with schizophrenia?

**DOI:** 10.1186/s12888-022-04510-0

**Published:** 2023-01-17

**Authors:** Alona Kaizerman-Dinerman, David Roe, Naor Demeter, Naomi Josman

**Affiliations:** 1 Department of Occupational Therapy, Faculty of Social Welfare & Health Sciences, University of Haifa, Haifa, Israel; 2 Department of Community Mental Health, Faculty of Social Welfare & Health Sciences, University of Haifa, Haifa, Israel

**Keywords:** PANSS, Positive and negative syndrome scale, Symptom, Moderator, Metacognitive limitation, Participation

## Abstract

**Background:**

Literature explains participation limitations among people with schizophrenia through the context of metacognitive limitations, specifically in symptoms and in executive functions (EF). Research has shown mixed results regarding associations between symptoms and participation, reporting association with negative symptoms, positive symptoms, or only metacognitive limitations. The aim of this study was to deepen understanding of the symptoms’ impact on the association between participation and executive function among people with schizophrenia.

**Methods:**

Forty-three participants with schizophrenia received 8 group sessions of focused metacognitive intervention (MCG) aimed at promoting participation by focusing on EF components (e.g., analyzing individual cognitive strategy use). Three measures were administered: the Positive and Negative Syndrome Scale (PANSS) to evaluate symptoms, the Weekly Calendar Planning Assessment (WCPA) to assess EF, and the Activity Card Sort (ACS) to measure participation at the baseline and 12 weeks following completion of the intervention. Scores were compared to a matched control group of 41 people with schizophrenia who instead received treatment as usual. The role of PANSS as moderator was examined using multiple hierarchical regressions, entering interactions between the PANSS scores and WCPA change scores in the final regression step.

**Results:**

Relationships were not significant for participants with high PANSS scores. A positive relationship existed between change in WCPA and change in ACS for participants with low PANSS scores.

**Conclusions:**

These results demonstrate that low PANSS scores moderate the association between EF and participation and highlight the importance of symptoms as a predictor of participation following the MCG intervention.

**Trial registration:**

The trial was retrospectively registered at clinical.trial.gov. ClinicalTrials.gov Identifier: NCT05556941. Clinicaltrial.gov registration date: 27/09/2022.

## Background

Participation, a term used within the international classification of functioning, disability, and health [1], is active engagement in everyday life, family, work, and community. It results from interactions among factors related to the person (e.g., metacognitive functions) and the person’s environment and chosen activity or occupation [2–4].

Research has examined the relationships between participation, metacognitive functions, and symptoms among people with schizophrenia [5–7]. Metacognitive functions represent goal-oriented behaviors, including decision-making, flexibility of thought, insight, and judgment [8]. Metacognitive function is a higher-level cognitive function that includes accurately judging task demands, anticipating the likelihood of problems, and monitoring, regulating, and evaluating performance within the activity’s context [9, 10]. Metacognition is also viewed as an umbrella concept that includes awareness and executive functions (EF) [11, 12].

Metacognitive functions are considered specific predictors of functional limitations that manifest in reduced participation [5, 13]. Specifically, symptoms [14] were negatively associated with limitations in metacognitive functions among people with schizophrenia in contrary to nonclinical populations [15]. Therefore, the impact of metacognitive limitations on participation is of essential clinical and theoretical interest.

Schizophrenia symptoms are typically measured by the Positive and Negative Syndrome Scale (PANSS) [16]. The appearance of symptoms also might partly explain reduced participation. However, the literature revealed mixed results regarding the association of symptoms with EF limitations and their impact on participation. For example, alongside MacBeth et al.’s study identifying associations between metacognitive limitations and symptomatic distress [17], researchers have reported that both positive [18] and negative [19] symptoms specifically predicted participation.

Some studies found positive correlations between negative symptoms, cognitive limitations (e.g., attention and memory), and metacognitive limitations (e.g., EF); other studies reported negative correlations [20]. Further, associations have been found between positive symptoms and EF limitations [21], alongside studies that found no association with cognitive limitations in general or EF limitations in particular [22]. Moreover, researchers claimed that people with schizophrenia and the greatest metacognitive limitations also manifested the most pronounced negative symptoms. Thus, at least on a cross-sectional basis, metacognitive limitations are linked to psychiatric symptoms [23].

To explain the relationships among symptoms, EF, and participation, researchers explored whether those relationships are direct or due to associations with other factors [24]. For example, the person’s functional capacity [24], insight [25], and social cognition [26] have predicted the severity of everyday functioning limitations. A study on the ability to perform skills postulated that whereas negative symptoms affect the likelihood of functioning, EF affect the ability to function [26].

Various models in the literature address the association among symptoms, metacognitive limitations, and participation in people with schizophrenia. Lysaker et al. proposed an integrated metacognition model in which metacognitive limitations and negative symptoms mutually influence one another and contribute to reduced participation [27]. However, another meta-analytic review produced contradictory evidence: Negative symptoms were found to predict short-term functioning, whereas metacognition affected long-term functioning [5].

Other studies found that negative symptoms partially modulated the relationship between metacognitive function and participation [23, 28] – such as how motivation and anhedonia evolve during the illness progression and how each might contribute at different times [23]. Moreover, education predicted functional capacity, which supported claims that metacognitive limitations have both direct and indirect effects on participation [28].

Considering the range of inconsistent findings, it is necessary to explore the impact of symptoms on participation to gain a better understanding of the relationship between metacognitive limitations and symptoms and the mechanisms that affect participation [19, 29]. As such, we used measures with high ecological validity in our study.

Identifying moderators may help explain how or why symptoms influence participation. Thus, the aim of this study was to examine whether symptoms moderate the association between EF components and participation among people with schizophrenia following a metacognitive group intervention (MCG) [6]. We hypothesized that symptoms constitute a moderator between EF as a component of metacognition and participation among people with schizophrenia.

## Methods

### Participants

The final sample for this study was 84 participants who completed an efficacy study of a metacognitive intervention for people with schizophrenia [6]. That study included participants based on three inclusion and two exclusion criteria. To be included, they had to (1) speak Hebrew fluently, (2) be diagnosed with schizophrenia by a licensed psychiatrist, and (3) score above the recommended threshold for abnormal EF (at least 65) on the Behavior Rating Inventory of Executive Function-adult version (BRIEF-A) [30]. Any severe psychiatric hospitalization for more than 24 hr. in the previous month or use of alcohol or drugs excluded a potential participant from the study. Five participants did not complete the preintervention assessment, and five refused to continue in the study and were eliminated, bringing the sample total to 84.

The 84 participants were randomly assigned to an intervention (*n* = 43) or control (*n* = 41) group according to the order in which they joined the study. Based on their existing psychiatric disabilities, participants in both groups were already eligible for and used a variety of psychiatric rehabilitation services. However, because the intervention group participants attended the MCG intervention during the research period (and the control group did not), they received an additional 2 hr of treatment each week throughout the research period [6].

Table 1 summarizes the sample demographics. Fifty-three (63.1%) of the 84 participants were men, and 31 (36.9%) were women. They ranged in age from 23 to 68 yr. The highest education level was 15 yr (*M* = 10.83, *SD* = 2.19). Most (56%) were unmarried, and 37 (44.0%) lived independently.

**Table 1:** Demographic characteristics of study participants

Demographic		Total (%) (*N* = 84)	Intervention group (*n* = 43)	Control group (*n* = 41)	Difference
Sex	Male	53 (63.1)	26 (60.5)	27 (65.9)	*z* = 0.510 *p* = 0.600
	Female	31 (36.9)	17 (39.5)	14 (34.1)	
Family Status	Single	47 (56.0)	28 (65.1)	19 (46.3)	χ^2^(2) = 3.110 *p* = 0.210
	Married	21 (25.0)	9 (20.9)	12 (29.3)	
	Divorced/ widowed	16 (19.0)	6 (14.0)	10 (24.4)	
Housing	Independent	37 (44.0)	18 (41.9)	19 (46.3)	*z* = 0.410 *p* = 0.670
	Protected	47 (56.0)	25 (58.1)	22 (53.7)	
	Range	*M* (*SD*)			*t*(82)
Age	23–68 yr	46.85 (12.25)	43.19 (11.77)	50.68 (11.69)	2.930 *p* = 0.004
Education	6–15 yr	10.83 (2.19)	11.00 (1.94)	10.66 (2.44)	−0.710 *p* = 0.478

### Instruments

#### Screening instruments

##### PANSS

Was used to assess psychopathological dimensions. The PANSS includes 16 psychopathological, seven negative syndrome and seven positive syndrome items (total 30) [16]. The scores for each item’s severity are rated on a scale from 1 to 7, with 7 indicating the most severe. The general PANSS score, which indicates the state of the disease, ranges from 30 (*no symptoms*) to 210 (*very severe*). Total scores for general psychopathology can range from 16 to 112 and, for the positive and negative syndrome items, from 7 to 49. The PANSS showed satisfactory internal reliability (psychopathological, α = .89; negative syndrome, α = .88; positive syndrome, α = .86) in this study.

##### BRIEF-A

A structured questionnaire was used to assess executive functions, the BRIEF-A consists of 75 items rated on a scale of 1 to 3, with 3 indicating a behavior that occurs *very often*, 2 indicating it occurs *sometimes*, and 0 indicating *never* [30]. The BRIEF-A produces two index scores – the behavioral regulation and metacognitive indices – and an overall global executive composite score. Using comparisons with a normative sample of 1200 informants and 1050 self-reports, the standard global executive composite scores and *t* scores were calculated for each clinical scale (higher scores signify that the individual experiences more severe difficulties). A normative score of *t* = 50 is expected; scores above 65 signify clinical impairment. The BRIEF-A’s moderate-to-high internal consistency ranges between α = .58 and α = .92; it shows high test–retest reliability between *r* = 0.91 and *r* = 0.94 and high test–retest stability between *r* = 0.82 and *r* = 0.94 [31].

#### Outcome measure: Weekly Calendar Planning Activity (WCPA)

WCPA was used to assess functional cognitive performance. A performance-based measure that examines the influence of subtle EF difficulties on the ability to perform multistep activities in daily life, the Weekly Calendar Planning Activity (WCPA) [32] incorporates scheduling meetings into weekly activity planning. It has three versions (difficulty levels). For this study, we used the moderately difficult version 2, which includes assignment of 17 appointments and outcome measures of *number of correct answers*, *efficiency of strategies*, and *number of strategies*. The coding and performance interrater reliability scores ranged between .94 and .99, and the performance measure has shown moderate to high test–retest reliability (with interclass correlations between .60 and .85) [33]. Low-to-strong correlations were found between the WCPA and other EF measures [34, 35].

#### Activity card sort

The Activity Card Sort (ACS) [2], was used to assess participation. The ACS aims to determine the disease’s effect on a person’s participation and activity levels, comprises 89 pictures representing activity categories. Respondents identify each picture according to their participation level in that activity. Possible answers are 0 (*did not do prior to the illness*), 0.5 (*beginning to do again*) or 1 (*did prior to illness and do now*). The final score determines the percentage of change in activity since disease onset. The ACS exhibits high internal consistency for sociocultural activities (α = .80) and instrumental activities of daily living (IADL; α = .82) but lower consistency for physical leisure activities (α = .66 for low physicality and α = .61 for high physicality) [36].

### Procedure

The Faculty of social welfare and health sciences in the University of Haifa Ethics Committee (271/14) and the Ministry of Health approved the study protocol, and all participants signed informed consent forms. An occupational therapist screened both groups two weeks before the begging of the intervention and evaluated participants in both groups before the intervention. Another occupational therapist (blinded to participants’ group membership) evaluated participants 4 weeks (after the intervention) and 12 weeks (follow-up) later. Three occupational therapists who had not performed the prior evaluations conducted the interventions. To verify the occupational therapists’ adherence to the protocol, the researcher provided them with 8 hours of study-specific training before the intervention and supervised each for 2 hours weekly during the intervention.

### Data analysis

We examined pre-intervention group differences and gender differences in study variables using iterative *t* tests, calculated Pearson correlations between the study variables and participants’ ages and education and defined standardized adjusted residual gains for the ACS and the WCPA. A repeated measures ANCOVA was used to examine the group difference for change in ACS, with age as the covariate. Thereafter, we conducted a repeated measures MANCOVA for the WCPA by both time and group and calculated Pearson correlations for the adjusted residual gains of the ACS, WCPA, and initial PANSS scores. We examined the PANSS for a moderating role in relationships between changes in the WCPA and ACS using the Process procedure.

## Results

### Preliminary results

Pre-intervention group differences were non-significant for the PANSS and WCPA but significant for the ACS, *t*(82) = 4.93, *p* < .001. The intervention group scored higher (*M* = 4.52, *SD* = 1.64) than the control group (*M* = 2.93, *SD* = 0.31).

The ACS at follow-up was negatively related to the participants’ ages (*r* = −.24, *p* = .027), as was the WCPA post-intervention (*efficiency r* = −.25, *p* = .020; *correct answers r* = −.29, *p* = .008) and at follow-up (*strategies r* = −.31, *p* = .004; *correct answers r* = −.36, *p* = .001). Thus, the participants’ ages were controlled for throughout the analyses. Gender differences and correlations with years of education were not significant.

### Main results

**Fig. 1:**
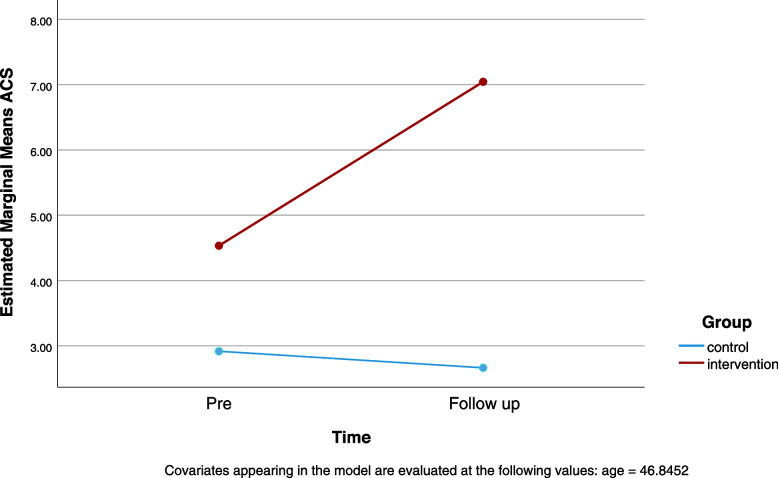
*Repeated measure MANCOVA for ACS group-by-time interaction. Note.* MANCOVA = multivariate analysis of covariance; ACS = Activity Card Sort

The mean PANSS positive was 14.33 (*SD* = 5.91, range 7–49), mean PANSS negative was 19.83 (*SD* = 5.31, range 7–49), and mean PANSS psychopathology was 31.92 (*SD* = 8.98, range 16–112). As shown in Table 2, repeated ANCOVA for the ACS revealed a significant group-by-time interaction with a high effect size)η_p_^2^ = .30). A significant increase was noted in the intervention group (*p* < .001) with a high effect size (η_p_^2^ = .43), but no change was evidenced in the control group. To examine the significant pre-intervention group difference in ACS score, a variable of “ACS difference” was calculated by the formula: post ACS score - pre ACS score/ pre ACS. When controlling the pre intervention ACS difference; ANCOVA test showed a significant group differences in “ACS difference” [F (1,81) =13.35, *p* < .001, η_p_^2^ = .142]. Figure 1 shows the ACS group-by-time interaction.

**Table 2:** Intercorrelations among ACS and WCPA change scores and PANSS

Instruments	2	3	4	5	6	7
1. ACS	.57***	.49***	.57***	−.07	.03	−.12
2. WCPA: Efficiency		.46***	.83***	.10	.06	.09
3. WCPA: Strategies			.53***	.15	.10	.14
4. WCPA: Correct answers				.10	.15	.18
5. PANSS: Positive					.34**	.70***
6. PANSS: Negative						.68***
7. PANSS: Psychopathology						

*N =* 84; *ACS* Activity Card Sort, *WCPA* Weekly Calendar Planning Activity, *PANSS* Positive and Negative Syndrome Scale

**p < .01, ***p < .001

Repeated MANCOVA measures of the WCPA by group and time revealed significant group-by-time interactions with moderate effect sizes. In all cases, significant increases were noted in the intervention group with high effect sizes: *efficiency*, *F*(2, 80) = 69.42, *p* < .001, η_p_^2^ = .64; *strategies*, *F*(2, 80) = 29.95, *p* < .001, η_p_^2^ = .43; and *correct answers*, *F*(2, 80) = 86.27, *p* < .001, η_p_^2^ = .68. Regarding *efficiency of strategies*, a significant increase was noted in the scores from pre- to post-intervention (*p* < .001) and in stability from post-intervention to follow-up (*p* = .173). For *number of strategies*, a significant increase was noted in the scores from pre- to post-intervention (*p* < .001), and another significant increase between post-intervention and follow-up (*p* = .010). Finally, for *number of correct answers*, a significant increase was noted in scores from pre- to post-intervention (*p* < .001), and another significant increase from post-intervention to follow-up (*p* = .034). All time differences in the control group were non-significant.

Change scores for the study outcomes (pre-intervention to follow-up) were calculated with adjusted residual gains (standardized), controlling for the initial score. Intercorrelations between these change scores and the PANSS (Table 3) revealed significant relationships. Increases in the WCPA and ACS scores were interrelated but unrelated with the initial PANSS scores.

**Table 3:** Variable means, standard deviations, and F-values by group and time

Measure	Intervention group (*n* = 43) *M (SD)*			Control group (*n* = 41) *M (SD)*			*F*_(1, 81)_ (η_p_^2^)	*F*_(1, 81)_ (η_p_^2^)
	Pre-intervention	Post-intervention	Follow-up	Pre-intervention	Post-intervention	Follow-up	Time	Time x group
ACS	4.52 (1.64)	–	7.06 (2.58)	2.93 (0.31)	–	2.65 (1.35)	2.41 (.029)	34.08*** (.30)
WCPA							*F*_(2, 160)_ (η_p_^2^)	*F*_(2, 160)_ (η_p_^2^)
Efficiency	0.34 (0.26)	0.56 (0.36)	0.60 (0.35)	0.31 (0.11)	0.29 (0.11)	0.27 (0.11)	3.04 (.037)	30.69*** (.277)
Strategies	2.49 (0.91)	3.28 (0.80)	3.58 (1.03)	2.56 (0.74)	2.54 (0.60)	2.56 (0.67)	1.74 (.021)	22.17*** (.217)
Correct answers	6.77 (2.28)	8.51 (2.77)	9.16 (2.84)	6.98 (1.78)	6.68 (1.63)	6.63 (1.85)	2.35 (.029)	29.89*** (.272)

*N* = 84; Age as covariate

***p < .001

To assess the PANSS’s moderating role in the relationship between changes in the WCPA and changes in the ACS, nine Process procedures were conducted. Age was entered as a control variable; independent variables were represented by each WCPA dimension, and mediators were represented as each PANSS score (Table 4). Results revealed that five of nine interaction terms were significant.

**Table 4:** PANSS as a moderator between change in WCPA and change in ACS

*Moderator (Mod): PANSS negative independent variable (IV): WCPA strategies*						
	Coefficient	*SE*	*t*	*p*	LLCI	ULCI
Constant	.1392	.4982	.2794	.7807	−.8524	1.1308
WCPA strategies	1.7698	.4146	4.2692	.0001	.9447	2.5950
PANSS negative	.0111	.0172	.6455	.5205	−.0231	.0453
Int_1	−.0587	.0185	−3.1750	.0021	−.0955	−.0219
Age	−.0074	.0075	−.9924	.3240	−.0224	.0075
*Mod: PANSS positive IV: WCPA efficiency*						
	Coefficient	*SE*	*t*	*p*	LLCI	ULCI
Constant	.5826	.4447	1.3102	.1940	−.3027	1.4679
WCPA efficiency	1.0219	.2287	4.4686	.0000	.5666	1.4772
PANSS positive	−.0228	.0151	−1.5026	.1370	−.0529	.0074
Int_1	−.0323	.0149	−2.1670	.0333	−.0619	−.0026
Age	−.0050	.0076	−.6494	.5180	−.0201	.0102
*Mod: PANSS Psychopathology IV: WCPA correct answers*						
	Coefficient	*SE*	*t*	*p*	LLCI	ULCI
Constant	.8730	.4639	1.8818	.0635	−.0504	1.7963
WCPA correct answers	1.3354	.294	4.5429	.0000	.7503	1.9206
PANSS psychopathology	−.0176	.0101	−1.7475	.0844	−.0376	.0024
Int_1	−.0246	.0093	−2.6362	.0101	−.0432	−.0060
Age	−.0058	.0073	−.7930	.4302	−.0204	.0088
*Mod: PANSS Psychopathology IV: WCPA efficiency*						
	Coefficient	*SE*	*t*	*p*	LLCI	ULCI
Constant	.7019	.4812	1.4588	.1486	−.2560	1.6598
WCPA efficiency	1.2837	.3117	4.1187	.0001	.6632	1.9042
PANSS psychopathology	−.0119	.0102	−1.1630	.2484	−.0322	.0085
Int_1	−.0239	.0101	−2.3785	.0198	−.0440	−.0039
Age	−.0064	.0076	−.8388	.4042	−.0215	.0088
*Mod: PANSS Psychopathology IV: WCPA strategies*						
	Coefficient	*SE*	*t*	*p*	LLCI	ULCI
Constant	.8674	.4822	1.7988	.0759	−.0924	1.8272
WCPA strategies	1.8595	.4280	4.3446	.0000	1.0076	2.7114
PANSS psychopathology	−.0122	.0100	−1.2148	.2281	−.0322	.0078
Int_1	−.0400	.0123	−3.2472	.0017	−.0645	−.0155
Age	−.0095	.0073	−1.3027	.1965	−.0241	.0050

*N =* 84, *PANSS* Positive and Negative Syndrome Scale, *WCPA* Weekly Calendar Planning Activity, *Int_1* Interaction effect, *LLCI* Lower limit confidence interval, *ULCI* Upper limit confidence interval

**Fig. 2:**
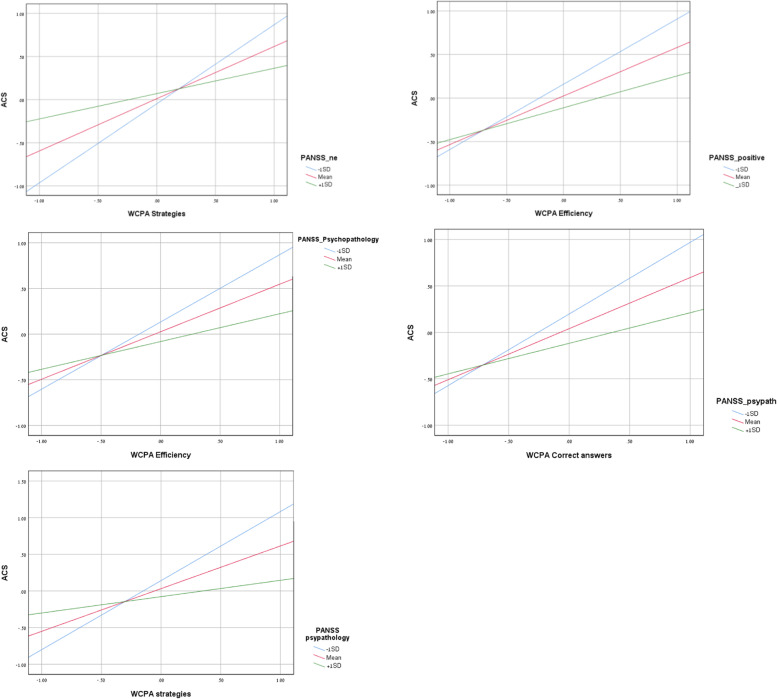
*PANSS as a Moderator between Change in WCPA and Change in ACS. Note.* PANSS = Positive and Negative Syndrome Scale; WCPA = Weekly Calendar Planning Activity; ACS = Activity Card Sort

These findings show that for low and mean PANSS scores (positive, negative, and psychopathology), a positive relationship existed between the change in WCPA (*efficiency*, *strategies*, and *correct answers*) and the change in ACS. For high PANSS scores, the relationship was non-significant. Specifically, the PANSS negative was a moderator between change in WCPA strategies and change in ACS. The PANSS positive was a moderator between change in WCPA efficiency and change in ACS. The PANSS psychopathology was a moderator between change in WCPA strategies, efficiency, and correct answers and change in ACS (Fig. 2).

## Discussion

This study investigated the impact of schizophrenia symptoms on the association between EF and participation. The results indicate that low and mean PANSS scores (positive, negative, and psychopathology) moderate the relationship between the change in the WCPA outcomes (*efficiency of strategy*, *number of strategies*, and *number of correct answers*) and the change in the ACS scores: For low and mean PANSS scores, there were positive relationships between EF components and participation; for high PANSS scores, the relationships were not significant.

These results may shed light on the inconsistent associations found in previous studies between symptoms, metacognition, and participation. Whereas this study addresses the symptoms’ severity and characteristics, most previous studies related to the symptoms’ (positive or negative) nature and impact. Rollins et al. found differences in the impact of positive and negative symptoms on coping-strategy types and functional outcomes among people with schizophrenia [37]. Those authors explained that positive symptoms tended to be episodic and had no significant effect on function, whereas negative symptoms constituted a stable phenomenon and had a profound effect on daily functioning.

Similarly, in their meta-analysis, Ventura et al. found that only negative symptoms were associated with functional limitations and they mediated, at least partly, the association between cognitive limitations and function [7]. However, the relation to positive symptoms was relatively weak. Ventura et al. explained that negative symptoms and cognitive limitations share many features, such as a similar type of onset. Positive symptoms, however, do not consistently interfere with a person’s ability to function (e.g., work). Thus, a person might learn to compensate for positive-symptom deficits in various ways. On the other hand, negative symptoms might relate more closely to functional limitations and skill acquisition. Studies showed differences in the impact of positive and negative symptoms on metacognition and functional outcomes among people with schizophrenia [26, 38]. Our study’s results link symptoms to the components of knowledge (metacognition) and performance (participation). This is consistent with Harvey and Strassnig’s study, in which they explained that symptoms might affect a person’s *likelihood* to perform skills, and metacognitive limitations may affect their ability to *perform* those skills [26].

Most studies that addressed and explored the unique symptoms of people with schizophrenia used the PANSS. Our study goes a step further, providing insight into the symptoms by distinguishing between their severity and characteristics. Similarly, Leutwyler et al. studied multiple factors that contribute to poor health among older adults with schizophrenia and found associations among symptoms of greater severity, poorer cognitive outcomes, and lower physical activity levels [39].

In addition, the literature has shown that people with schizophrenia experience difficulties with EF, which affects their independence and participation levels in IADL [40]. To assess clients’ functional abilities within IADL adequately and identify their strengths and weaknesses, assessment methods should simulate real-life activities and capture nuanced performance during complex tasks [41]. Such results can yield information on the real-life performance of EF, which is important for clinicians’ and researchers’ understanding of how EF manifest in community living [42].

Our results support the use of the WCPA as an easy-to-use, ecologically valid measure of EF among people with schizophrenia. This type of assessment coincides with the broader definition of health and wellness conceptualized in the international classification of functioning disability and health, which recommends including measures for independence and participation in daily life activities [1]. As far as we know, this study is the first to use the WCPA to measure the improvement during a metacognitive intervention for people with schizophrenia [6, 43].

Our findings also have implications for interventions. Cognitive interventions have been criticized because cognition improvements typically do not generalize to daily life. This study’s results suggest symptoms moderate between EF and participation among people with schizophrenia. Therefore, symptom severity should be assessed before implementing cognitive interventions to maximize participation.

### Future research

Our results support the use of the WCPA as an ecologically valid measure of EF among people with schizophrenia. Although our findings support the moderating role of the PANSS, other moderators or mediators likely exist. For example, information processing can support the efficient use of strategies and enhance participation [44, 45]. We recommend future studies use more assessments, such as the Measurement and Treatment Research to Improve Cognition in Schizophrenia, which is considered an endpoint in clinical trials aimed at alleviating neurocognitive impairments in schizophrenia. Such alternative moderators should be considered when determining predictors of participation. It is also recommended to consider using the CAINS [46] to better distinguish the expressive component of negative symptoms. Further research is needed to identify these variables and the efficacy of MCG interventions that target EF and participation. It is important to address the PANSS score so that participants can benefit more from MCG interventions aimed at broadening participation and daily functioning. Future studies also need to elaborate on and strengthen these findings.

### Study limitations

This study had some methodological limitations that warrant consideration. First, we assigned participants to the intervention or control group according to their order of arrival rather than strict group randomization, which might limit generalization of our findings. In addition, there was no group matching for age. Thus, future studies are needed to cross-validate our findings in the same population. Second, we used a single-referral source (a professional support center), which might bias the sample regarding the PANSS severity in some way. The possibility of low heterogeneity in repeated studies should be addressed. The PANSS does not reflect the new generation scale like CAINS which specifically focuses on distinguishing the expressive component of negative symptoms. In addition, the WCPA focuses on evaluating only EF, but the cognition and metacognition concepts are broader than just EF.

## Conclusions

This study explored and helped provide a better understanding of the moderating role of symptom severity in the relationship between EF and participation. Evaluating this relationship can be useful in determining which underlying mechanisms influence functional outcomes and can help clinicians design symptom-management strategies specifically for people with schizophrenia.

## Data Availability

The datasets used and/or analysed during the current study available from the corresponding author on reasonable request.

## Abbreviations

ACS: Activity Card Sort
BRIEF-A: Behavior Rating Inventory of Executive Function-adult version
EF: Executive functions
IADL: Instrumental activities of daily living
MCG: Metacognitive group
PANSS: Positive and Negative Syndrome Scale
WCPA: Weekly Calendar Planning Activity
